# Primary production ultimately limits fisheries economic performance

**DOI:** 10.1038/s41598-021-91599-0

**Published:** 2021-06-16

**Authors:** Anthony R. Marshak, Jason S. Link

**Affiliations:** 1 CSS, Inc. in Support of NOAA’s National Centers for Coastal Ocean Science, National Ocean Service, National Oceanic and Atmospheric Administration, Silver Spring, MD USA; 2 National Oceanic and Atmospheric Administration, National Marine Fisheries Service, Office of the Assistant Administrator, Woods Hole, MA USA

**Keywords:** Ecosystem ecology, Marine biology, Ocean sciences, Environmental social sciences, Environmental economics, Sustainability

## Abstract

Living marine resources (LMRs) contribute considerably to marine economies. Oceans continue to respond to the effects of global change, with environmental factors anticipated to impact future seafood production and its associated economic performance. Here we document novel relationships between primary productivity and LMR-based economics for US regional marine ecosystems and 64 international large marine ecosystems (LMEs). Intermediate relationships between production, total biomass, fisheries landings, revenue, and LMR-based employment are also elucidated. We found that all these factors were dependent on the amount of basal production in a given system. In addition, factors including human population, exploitation history, and governance interventions significantly influenced these relationships. As system productivity plays a foundational role in determining fisheries-based economics throughout global LMEs, greater accounting for these relationships has significant implications for global seafood sustainability and food security. Quantifying the direct link between primary production and fisheries economic performance serves to better inform ecosystem overfishing thresholds and their economic consequences. Further recognition and understanding of these relationships is key to ensuring that these connections are accounted for more effectively in sustainable management practices.

## Introduction

In both the United States (US) and globally, living marine resources (LMRs) and associated fisheries are important contributors to ocean economies^1–3^. Total LMR-related revenue and employment have increased over the past decades, with US marine fisheries currently valued at 210 billion USD and contributing on average ~ 2.5% of US ocean gross domestic product (GDP)^3,4^. Fisheries economic production is limited by factors including ecosystem-level constraints that have not been fully explored in past investigations^1–9^. As oceans continue to undergo global change, environmental and ecological factors are anticipated to become more limiting on seafood production and affect marine economies^2,10^. Addressing these future challenges will require broader management approaches that consider both marine ecosystem dynamics and human dimensions in concert^3,11–13^. Here we examine these socio-ecological relationships more closely for both US and all International Large Marine Ecosystems (LMEs)^3,8^.

Seminal works have addressed the value and sustainability of natural capital, including its connections to multiple marine ecosystems^14^, but have not always examined limiting factors. Previous studies have determined that sustainable fisheries harvest is related to the level of primary production (i.e., basal organic production) available within a given ecosystem^6,15,16^, with this limitation being a primary consideration when accounting for ecosystem overfishing^6,17,18^. Investigations in estuarine and lacustrine environments have similarly quantified the influence of nutrient loading (which impacts primary production) on fish production, with some hinting at ultimately limiting the magnitude of fisheries economics^19,20^. Yet there have been no examinations of the relationship among fisheries economic performance and primary productivity for marine waters. Theoretical studies, marine ecosystem models, and empirical examinations have estimated transfers of primary and higher-order production to biomass throughout trophic levels, identified shifts and perturbations in trophic composition, and demonstrated how LMR production and associated fisheries landings are ultimately limited by ecosystem production^3,6,15–18,21^. These relationships have also been shown to differ among regions, and are influenced by multiple human-related and environmental factors^2,3,6,15–18,21,22^. In this study, we detail these connections between primary productivity and economic performance at the LME scale.

## Results

**Figure 1:**
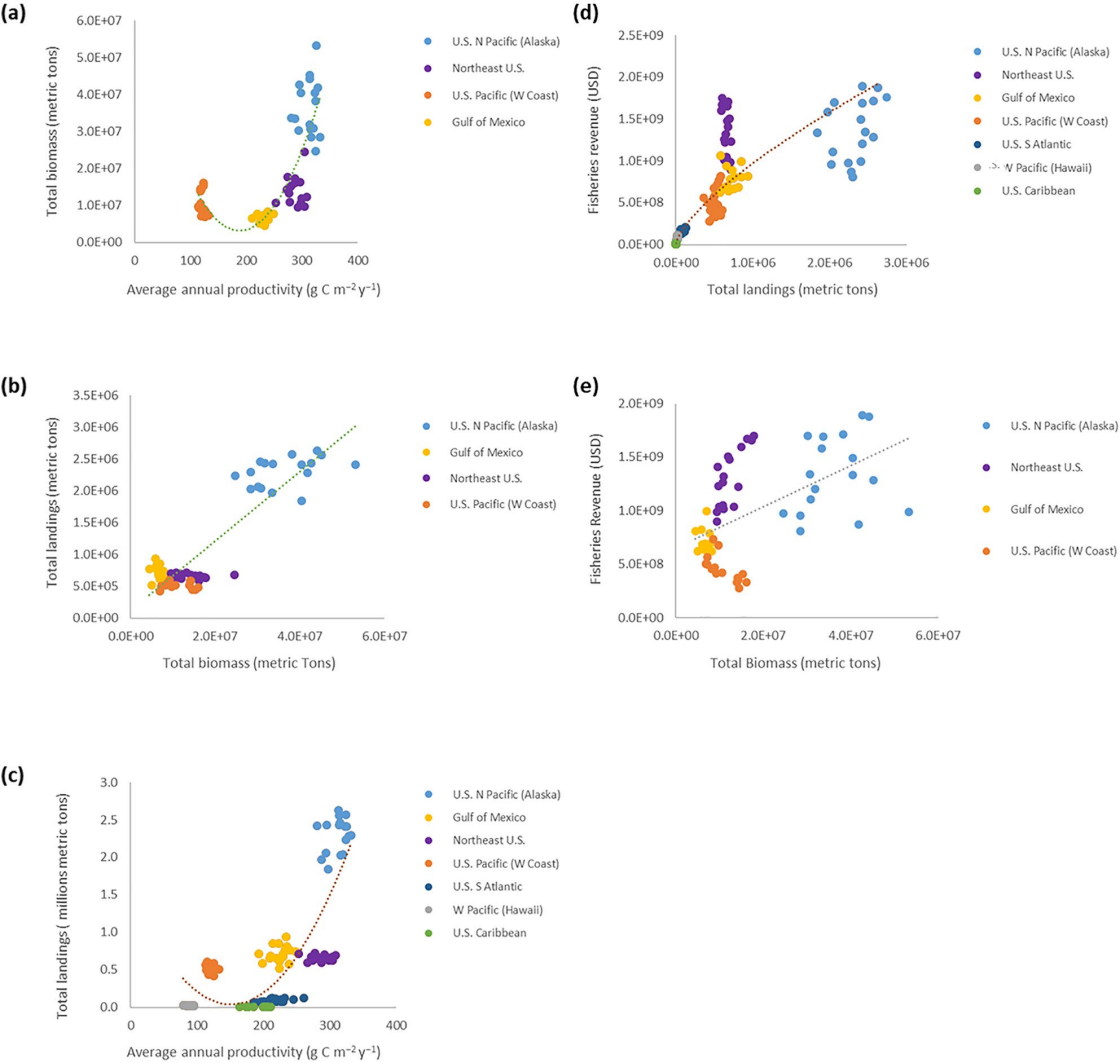
Throughout major US regions, relationships among primary productivity, surveyed biomass, fisheries landings, and total fisheries revenue. (**a**) Relationship between average annual primary productivity (g C m^−2^ year^−1^) and total surveyed fish and invertebrate biomass (metric tons; y = 1742.4x^2^ − 6.6 × 10^5^x + 6 × 10^7^; r^2^ = 0.642; p < 0.0001). (**b**) Relationship between total surveyed fish and invertebrate biomass (metric tons) and total fisheries landings (metric tons; y = 0.0547x + 111,982; r^2^ = 0.813; p < 0.0001). (**c**) Relationship between average annual primary productivity (g C m^−2^ year^−1^) and total fisheries landings (metric tons; y = 66.092x^2^ − 20015x + 2 × 10^6^; r^2^ = 0.667; p < 0.0001)﻿. (**d**) Relationship between total fisheries landings (metric tons) and total fisheries revenue (USD; y = 32187x^2^ − 8 × 10^6^x + 7 × 10^8^; r^2^ = 0.600; p < 0.0001). (**e**) Relationship between total biomass (metric tons) and total fisheries revenue (USD; y = 19.282x + 6 × 10^8^; r^2^ = 0.293; p < 0.0001). Data cover years 1998–2014.

When investigating these relationships more closely, we find strong positive relationships in US LMEs for total fisheries landings as a function of average primary productivity rates (Fig. 1c). Highest landings during this period (years 1998–2014) were observed in the most productive US regions (i.e., US North Pacific, Gulf of Mexico, and the Northeast US), while lower landings were associated with less productive tropical and subtropical areas. While other environmental factors, governance and management interventions, previous exploitation, and living marine resource status all affect regional fisheries landings^2,3,10,23,24^, primary production accounts for a major, foundational component of this variability (Fig. 1c, Table 1)^3,7–9,15^.

**Table 1:** Multiple regression relationships among independent and dependent variables within all US regions.

Independent variable	Dependent variable				
	Biomass	Landings	Revenue	LMR Jobs	% LMR Jobs
Primary productivity	**0.002 (+)**	**< 0.0001 (+)**	**< 0.0001 (+)**	**< 0.0001 (+)**	**< 0.0001 (+)**
Biomass	–﻿	**< 0.0001 (+)**	**< 0.0001 (+)**	**0.001 (−)**	**< 0.0001 (+)**
Landings	0.050﻿ (+)	–	**< 0.0001 (+)**	**< 0.0001 (+)**	**< 0.0001 (+)**
Revenue	–	–	–	**< 0.0001 (+)**	**< 0.0001 (+)**
Human population density	**< 0.0001 (−)**	**< 0.0001 (−)**	**< 0.0001 (−)**	**< 0.0001 (+)**	**< 0.0001 (−)**
Area of shelf	0.126 (+)	**< 0.0001 (+)**	**< 0.0001 (+)**	**< 0.0001 (−)**	**0.003 (+)**
Fishing effort (kwsd)	–	0.650 (+)	0.913 (+)	–	–
Landings/shelf area	NR (−)	–	0.930 (+)	NR (+)	NR (+)
% Stocks undergoing overfishing^a^	− 0.159 (−)	**< 0.0001 (−)**	**< 0.0001 (−)**	**< 0.0001 (−)**	**< 0.0001 (−)**
%Overfished stocks^a^	NR (−)	**< 0.0001 (−)**	**< 0.0001 (+)**	0.434 (+)	**0.044 (+)**
% Stocks overfishing status unknown^a^	NR (−)	**< 0.0001 (−)**	0.135 **(−)**	**0.001 (+)**	**0.001 (−)**
% Stocks overfished status unknown^a^	NR (+)	**< 0.0001 (−)**	**< 0.0001** (−)	**< 0.0001 (−)**	0.900 (+)
Total FMP interventions^a^	NR (+)	NR (+)	NR (+)	NR (+)	**0.002 (+)**
%EEZ permanently protected from fishing^a^	NR (+)	NR (+)	NR (+)	NR (−)	NR (+)
**Regression summary variables (with biomass)**					
R^2^	0.904	0.990	0.938	0.960	0.952
N	61	36	36	33	33
df	38	13	9	6	6
**Regr. summary variables (without biomass)**					
R^2^	–	0.993	0.975	0.992	0.971
n	–	63	63	61	61
df	–	40	38	36	36

p-values and sign of Pearson correlation coefficient value (i.e., + or − in parenthesis) are shown for relationships among independent variables and each dependent variable within ocean economies. Summary variables for each multiple regression relationship (R^2^, n, df) among a given dependent variable and all independent variables are included. Values are shown for regressions that include biomass (and without) to account for biomass estimates not being available for each US region of interest examined. Bold values indicate statistically significant relationships (p < 0.05).

*EEZ* exclusive economic zone, *FMP* Fishery Management Plan, *kwsd* kilowatt sea days, *NR* not reported.

^a^Indicates that only values from 2017 were used. Primary productivity reported as g Carbon m^−2^ year^−1^.

**Figure 2:**
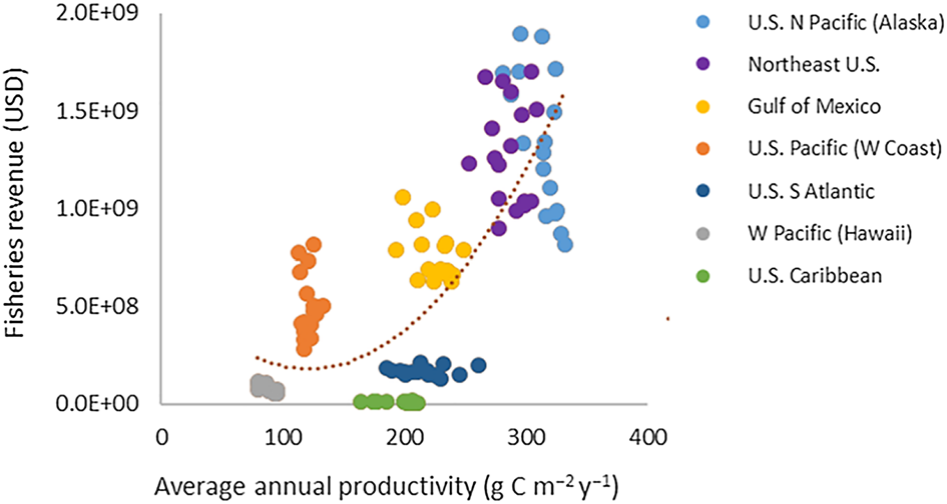
Relationship between average annual productivity (g C m^−2^ year^−1^) and total fisheries revenue (USD) throughout all major US regions. Years 1998–2014. Regression (y = 32187x^2^ − 8 × 10^6^x + 7 × 10^8^; r^2^ = 0.600; p < 0.0001).

**Figure 3:**
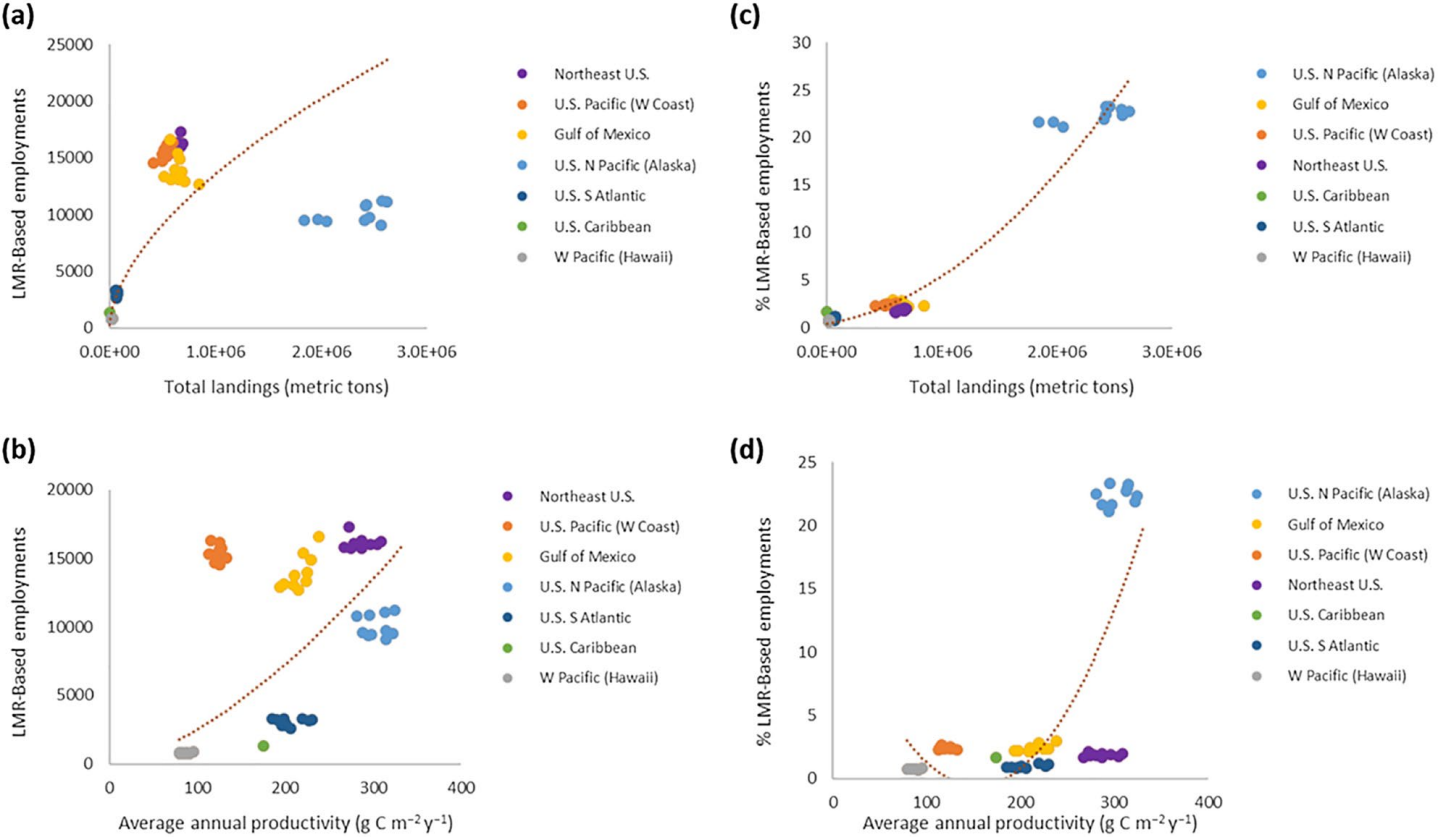
Throughout all major US regions, relationships between living marine resource (LMR)-based employment and percentage of LMR-based employment within the total regional ocean economy as related to total fisheries landings and primary productivity. (**a**) Relationship between total fisheries landings (metric tons) and total LMR-based employment (y = 4.6292x^0.5776^; r^2^ = 0.771; p = 0.006). (**b**) Relationship between total fisheries landings (metric tons) and total LMR-based employment (y = 2.206x^1.53^; r^2^ = 0.388; p < 0.0001). (**c**) Relationship between average annual primary productivity (g C m^−2^ year^−1^) and percentage of LMR-based employment within the total regional ocean economy (y = 3 × 10^−12^x^2^ − 2 × 10^−6^x + 0.3907; r^2^ = 0.957; p < 0.0001). (**d**) Relationship between average annual primary productivity (g C m^−2^ year^−1^) and percentage of LMR-based employment within the total regional ocean economy (y = 0.0006x^2^ − 0.1998x + 14.772; r^2^ = 0.533; p < 0.0001). Years cover 2005–2014.

In essence we illustrate that primary production influences total biomass (Fig. 1a), which positively affects total landings (Fig. 1b; c.f.^6,15–18^), reinforcing that primary productivity is also positively related to total landings (Fig. 1c). Total landings are related to fisheries revenue (i.e., fisheries performance; Fig. 1d), which is ultimately set by total biomass (Fig. 1e) and results in the main relationship that primary productivity is strongly related to total fisheries revenue (Fig. 2). Extending these associations to recognize the positive relationship between fisheries landings and LMR-based employment (Fig. 3a) implies that primary productivity can influence regional ocean employment (Fig. 3b), and percentages thereof for an entire regional ocean-based economy (Fig. 3c,d).

Annual fisheries revenue and LMR-based employment are highly dependent on the quantity (shown for US regions, Figs. 1d, 3a,c) and quality of landings^2–4,25^. Greatest US fisheries revenues over the past two decades have generally been observed in higher-latitude, productive systems, with values highest for the US North Pacific (i.e., Alaska), Northeast US, Gulf of Mexico, and US Pacific (i.e., West Coast) regions. Fisheries landings in the North Pacific are several times higher than in other US regions, while total revenue ranges have been generally comparable within North Pacific, Northeast US, and Gulf of Mexico systems (Figs. 1d, 2). Similarly, LMR-based employment is generally highest in these regions (Fig. 3a), although values are moderate in the more remote North Pacific due to the bulk of large volume species (i.e., pollock, Pacific cod, flatfishes) being captured by relatively low numbers of fleets and processors, and other contributing factors in that region such as weather and lower human population^3,4,26^. When considered in terms of the percentage of employment within a region’s total ocean economy, the North Pacific is overwhelmingly highest with LMR-based employment making up ~ 20–25% of its total oceanic workforce (Fig. 3c). These economic factors are likewise found to be dependent on the inherent basal productivity of a given regional ecosystem (Fig. 3c,d). While the relationship between productivity and total LMR-based employment is not as robust, factors including human population density (particularly in the North Pacific and US Pacific), exploitation history, shelf area, and fisheries revenue significantly influence these results (Table 1). In addition, the dominance of other ocean sectors, fishing effort and trip duration, and the revenue and operating costs associated with a given fisheries ecosystem all influence the percentage of LMR-associated employment in a given area, and thus its relationship with system productivity, biomass, and landings.

Although these connections have certainly been implied in the scientific literature, and may be inferred from past investigations^2–4,6,15,16,22,25–29^, this is the first documentation of the direct relationships between basal ecosystem productivity and LMR-based economic metrics. Examining these specific relationships within US LMEs suggests that their interconnectivity holds. We see that primary production does set much of the foundation for biomass in a given system over the same period of investigation in regions where total values are available (Fig. 1a), and that fisheries landings are likewise dependent on system-level biomass (Fig. 1b) and hence productivity (Fig. 1c). Although coral reef-based tropical systems such as those found in the Western Pacific follow an inverse trophic pyramid (i.e., greater upper-level trophic biomass in clearer, less productive waters) that has been subject to additional human disruptions^30^, and coastal upwelling systems like the US Pacific (West Coast) contain greater pelagic biomass supported by intermittently productive waters^31,32^, these productivity-biomass trends still generally persist. Observed parabolic relationships between productivity and biomass (Fig. 1a), landings (Fig. 1c), revenue (Fig. 2), and percentage of LMR-based employment were influenced by intermittent upwelling in the California Current ecosystem, which contributes to concentrated primary and secondary production that is spatiotemporally variable, including at depth. These patterns all generally hold when examining them on an areal basis (Supplementary Fig. 1) or at a total production level (Supplementary Fig. 2). Additional factors influence these variables (Table 1) and there are caveats to their proposed relationships. For example, many feedback loops are found in terms of system biomass and landings, with both having direct influences on each other^3,6,17,18,21–23,33,34^. While fisheries harvest is certainly dependent on available biomass, fishing additionally affects and can limit or alter biomass composition as classically observed in the Northeast US and other systems^3,6,17,18,21–23,35^. In addition, other contributors to primary production, including the deep chlorophyll layer, microbial loop, upwelling, trophic linkages, ecosystem turnover, and complementary processes can significantly affect these higher-order relationships^2,8,21,29,32,36–39^. Despite these caveats, the main patterns that fishery economic performances are derived from basal ecosystem production remain.

**Figure 4﻿:**
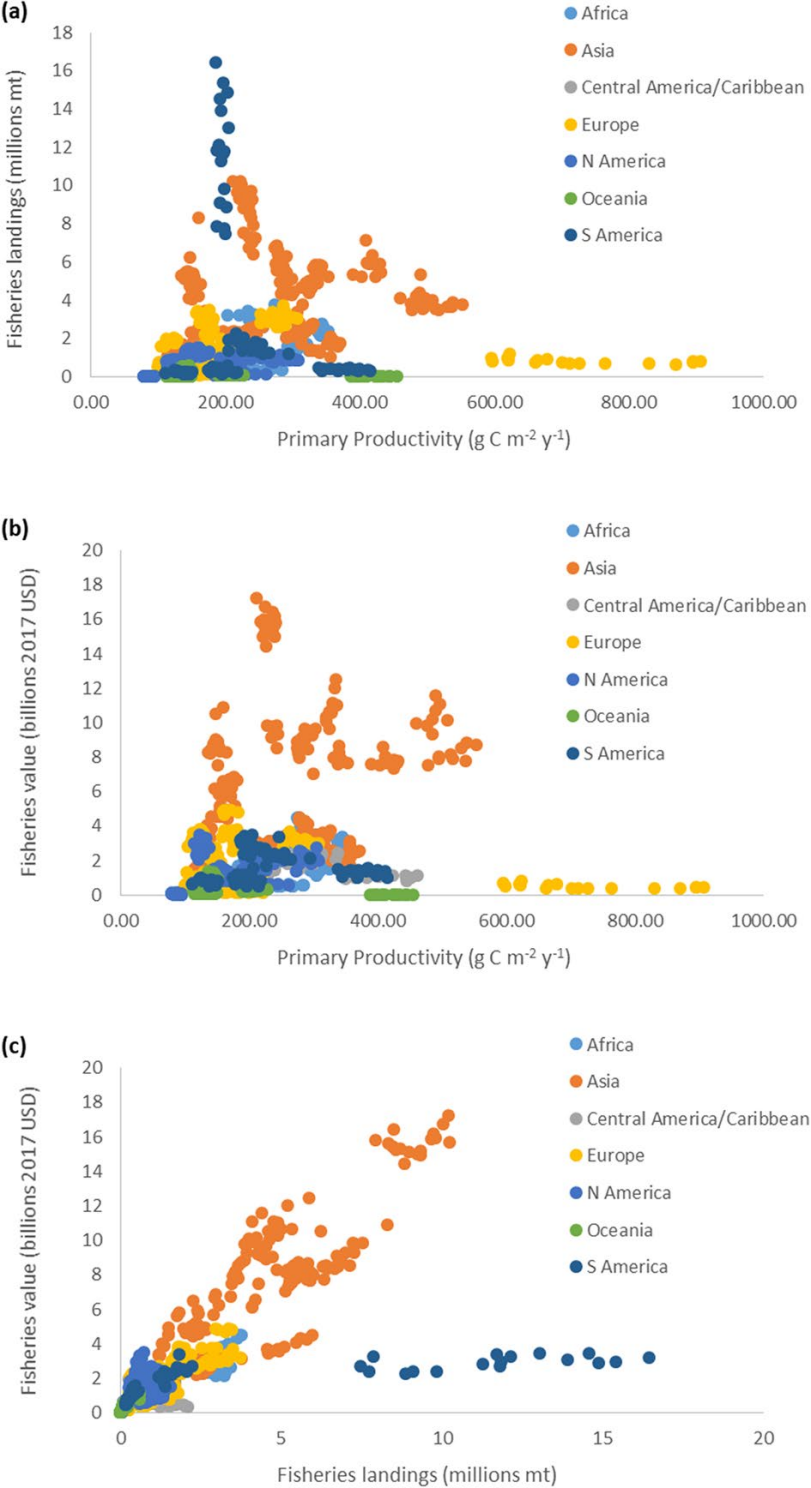
For 64 identified international Large Marine Ecosystems (as classified by continent), relationships among primary productivity, fisheries landings, and fisheries value. (**a**) Relationship between average annual primary productivity (g C m^−2^ year^−1^) and total fisheries landings (metric tons, mt). (**b**) Relationship between average annual primary productivity and fisheries value (2017 USD). (**c**) Relationship between total fisheries landings and fisheries value. Years cover 1998–2014.

While these patterns have been demonstrated for US marine ecosystems, particularly in temperate, sub-tropical and sub-polar geographies, we also explore how broadly these relationships hold for other marine ecosystems. Examining these relationships for 64 international LMEs, we confirm that these production-dependent economic patterns are repeated (Fig. 4a–c), with a few exceptions. Two main outliers include the eutrophic Baltic Sea^40^, where high productivities (associated with anthropogenic nutrient loading) and low landings and fisheries value (associated with overfishing)^41^ are observed (Fig. 4a,b, Supplementary Fig. 3a–c), and the Humboldt Current where high landings (with relatively low fisheries value) are found in an upwelling system with mid-range average production and low continental market value in South America for mostly small pelagic fisheries (Fig. 4a,c, Supplementary Fig. 3 g–i). In addition, low landings and revenue are associated with productive waters off the North Australian Shelf (Fig. 4a,b, Supplementary Fig. 3p,﻿q) due to historic exploitation, degraded ecosystem conditions, and potentially related to the increasing presence of marine protected areas^42,43^. Alternatively, highest landings and revenue in Oceania are found for the New Zealand Shelf, with mid-range productivity and where additional concerns have been highlighted regarding ecosystem condition (Supplementary Fig. 3p–r)^42^. In general, higher fisheries landings and revenue were observed off Asia, Europe, Africa, and South America, where mid to high productivities occurred (Fig. 4a–c, Supplementary Fig. 3). The salient point is that despite these caveated instances, the pattern of primary production establishing the magnitude of fishery economic performances is repeated globally.

## Discussion

If these relationships generally hold true throughout all LMEs, then significant implications for LMR-based economics and management follow. Chiefly, these results show that for any given marine ecosystem, its associated economic performance (i.e., fisheries revenue) is ultimately limited by inherent basal ecosystem productivity, as has been demonstrated for fisheries landings^15,16^. Therefore, within some systems we cannot expect their economic contributions to be as high (or to increase as readily) as in others due to the limitations of primary production. Ultimately, some regions are inherently more productive than others, which could translate into higher economic benefits that might be sustained over greater periods of time if exploitation rates remain at or below system thresholds^2,3,6,15,16,18,21,22,33,34^. In general, these relationships hold, but there are also outliers, which reinforce the importance of monitoring, understanding, and knowing the particular characteristics of each system. While regional LMR-based economies are also dependent on other human (including governance, exploitation, and market interventions) and environmental factors, their dependence on system-level primary production should be recognized more strongly, particularly in terms of limiting catch and LMR-based economics. Thus, currently productive systems in the US such as the North Pacific and Northeast US could see future climate-related changes to their basal productivities, which could extend to the sustainability of these major LMR-based economies^2,29,36–38^. The same would apply to other regions around the globe.

The global ramifications of these findings are not trivial, reinforcing studies that have highlighted concerns about the future sustainability of fisheries production and economics^2,10,29,36,38,44^. There is a foundational role that system productivity plays in determining ecosystem biomass, and both directly affect sustainable harvest rates and LMR-based economics throughout the majority of US and international LMEs. Specifically, greater accounting for the linkages between basal production, system-level biomass, landings, ecosystem overfishing, and LMR-based revenue and employment in management is integral for the successful future sustainability of marine fisheries and their dependent communities. This focus is especially warranted given that altered global primary production is predicted to occur as a result of ongoing climate change effects to the world’s ocean^2,29,36,38^. While these effects may not be observed for total, global oceanic primary production, future changes in the seasonality, distribution, and composition of the phytoplankton community may alter the productivity available to higher trophic levels within LMEs, with large ramifications for fisheries-associated economies^2,29,36–40^. In addition, continued natural and anthropogenic impacts to LMEs may lead to large-scale regime shifts, such as those currently observed for multiple systems, including the Baltic Sea, Benguela Current, California Current, and North Sea^40,41,45–47^. Redirected trophic flow and altered ecosystem structure would have significant effects on the primary production and biomass required to sustain catch, thereby affecting future harvest potential and its associated economics.

These relationships have particular application for ecosystem overfishing, where management of system-level landings, revenue, and LMR-based economics are considered relative to system productivity and biomass^3,6,12–14,17,18^. Efforts to prevent ecosystem overfishing, particularly in areas that have already exceeded thresholds (e.g., Northeast US, Gulf of Mexico, SE Asia, or some African LMEs)^3,6,33,34,48^ would wisely account for the relationships noted here under future management. These approaches also account for natural and human-related factors that influence system-level production, biomass, landings, and economics, whose characterizations are especially important in explaining trends and interrelationships in outlier ecosystems. As compounding natural and human-related stressors continue to affect marine ecosystems, and multiple fisheries stocks remain overfished or of unknown status, the urgency for these more holistic and adaptive approaches cannot be overstated.

Global fisheries value and employment continue to increase and have remained high over the past decades^2,29,37,49^. However, these trends are expected to change during the twenty-first century as a result of multiple factors, including climate-related production shifts, continued exploitation, and consequential increases in fishing-associated costs^2,29,37,44,48–51^. Global landings have effectively plateaued^44,49,51^, and together with their revenue are ultimately limited by primary production. While limited quantity and high demand may cause prices to increase by an order of magnitude for particular taxa in a given ecosystem, the associated maximum potential revenues and their variability are ultimately constrained by the amount of catch available, which itself is constrained by primary production. Accounting for these factors and their influences and greater commitment to monitoring global primary production, especially in under-resourced areas with high human population growth, is warranted. These relationships are especially worth monitoring for marine ecosystems that contain the most lucrative fisheries resources, and in those having greatest risks of ecosystem overexploitation; the estimation, validation, and assessment of primary production dynamics is a minor cost relative to the value of those fisheries.

Continuing to invest in broader, ecosystem-based approaches^3,6,11–13,52^, with strengthened understanding of the socioecological connections among marine ecosystems, natural phenomena, and human dimensions will allow for more thorough implementation and benefits of ecosystem-level approaches. To be able to consider these multiple factors together, a rethinking of how we view and approach both fisheries harvest and management is necessary. Efforts to shift from a traditional single-species focus to a more comprehensive ecosystem-based approach have been underway for several decades, with concrete implementation plans for US fisheries developed over the last few years^52^. The benefits of establishing maximum biomass removal caps, such as those for groundfish in the North Pacific^53^, continue to be observed. Their utility as a management tool for preventing ecosystem overfishing and ensuring sustainable LMR-based economics is reinforced by the findings of our study. The needs and benefits of ecosystem-based fisheries management are gaining in awareness, acceptance and implementation; these systematic marine management approaches better allow for the accounting of interconnected environmental, ecological, economic, and system-level tradeoffs^3,6,11–13,44,52^. Ultimately, greater recognition that foundational primary production within an ecosystem indeed limits its sustainable harvest, and thus the associated economic performance and benefits derived from its marine resources, is key to ensuring that these connections are accounted for more effectively in management approaches.

## Methods

To evaluate relationships between components of the pathway proposed by Link and Marshak^3^, from primary productivity, total surveyed fish and invertebrate biomass, fisheries landings, economic revenue, and living marine resource (LMR)-based employment for US regions (as defined by regional fishery management council and NOAA jurisdictions) and international LMEs, we used the following methods and data sources. Primary productivity estimates for each region or identified LME were measured by spatially characterizing annual regional primary productivity (g C m^−2^ year^−1^) and mean annual chlorophyll concentrations from NASA Ocean Color Web Data SeaWiFS years 1998–2007 and MODIS-Aqua years 2008–2014 (4 km resolution)^54^, using the Behrenfield and Falkowski Vertically Generalized Production Model (VGPM) estimation method^55,56^. Primary productivity values were averaged over published Large Marine Ecosystem (LME) areas^42^. These were then converted from units of Carbon to wet weight using a common scalar^6^. Nearshore benthic production throughout vegetated habitats (e.g., macroalgae, mangrove, salt marsh, seagrass) is important in some of these systems, but data are less comprehensively available as the extents of many of these areas are not well-mapped^57^. Given these limitations, and that the scale of this study occurred throughout the entire US EEZ, we did not incorporate benthic primary productivity estimates. Although performed in other studies that examine satellite data^58^, no additional correction for chlorophyll or productivity values were made for optically shallow waters.

During the same time period (1998–2014), annual estimates of total surveyed (demersal and pelagic) fish and invertebrate biomass were summed from NOAA Fisheries seasonal fishery independent surveys of most US regions^59,60^. Given surveying methodology constraints, total biomass was not estimable for the US Caribbean, US Western Pacific and US South Atlantic regions. Additionally, all productivity, landings, and economic data for the US Western Pacific region were limited to the Hawaiian Insular platform to correspond with the identified LME. Trends in total regional commercial and recreational landings (and as standardized by EEZ area; km^−2^) reported by NOAA Fisheries (https://www.fisheries.noaa.gov/national/sustainable-fisheries/commercial-fisheries-landings) were examined (years 1998–2014). For social and economic indicators, regional trends in total LMR employment (defined as the number of individuals working in LMR establishments), including their related percent contributions to the total oceanic workforce of a given region as defined and recorded by the National Ocean Economics Program^61^, were calculated per year over the past decades (years 2005–2014). Additionally, the total USD value of all commercially landed species as reported by NOAA Fisheries and regional FMCs was examined^4^ and normalized to the year 2017 to align with other variables (i.e., overfished/overfishing status, FMP interventions, percent EEZ protected) from that timeframe. We note that although more recent data are available, we stopped in 2014 to be consistent for data availability across all data sets (especially some of the international data) and to avoid any potential misinterpretation for current fishery management actions. Although landed highly migratory species are included in NOAA fisheries statistics for all regions, including the Western Pacific, the numbers and values are underestimated in light of the international jurisdictions of these species and records of capture beyond US waters throughout their range^62^. Thus, they are not used here and were considered to be low estimates.

Relationships for these metrics throughout the US EEZ were quantified using multiple non-linear and linear regressions; the form of regression chosen was that which had the lowest Akaike Information Criterion (AIC) and higher R^2^. Regressions were chosen to illustrate relationships among production, biomass, landings, and economic factors through easily understood, stepwise means that demonstrated the logic sequence of linked dependencies across those variables. Although generalized additive models (GAMs), mixed models (GAMMs), or multivariate approaches may allow for more detailed and integrated investigation of these relationships, they were not the primary means of analysis given our objective of establishing sequential relationships among the variables. And particularly with respect to possible multivariate statistical approaches the separate multiple regressions used to examine these relationships simultaneously accounted for all factors and covariates (with and without biomass included, due to biomass estimates only being available for four regions of interest) to examine the direct effect of a given independent variable (or covariate; n = 14) on a specific dependent variable (n = 5), and its significance (alpha = 0.05). In this analysis, the separate effects of landings on biomass, and of biomass on landings, in relation to relevant independent variables and covariates were examined. Annual total biomass, fisheries revenue, and LMR-based employment (including their percent contributions to a given ocean economy) for each region were examined as a function of primary productivity in all regions. Relationships were similarly examined between total biomass and fisheries landings, and for fisheries revenue and LMR-based employment as a function of landings. In addition, separate multiple regressions were employed to examine relationships among these dependent variables and ten other covariates from all US regions. Factors included human population density, continental shelf area for each US region, fishing effort, standardized landings per shelf area (km^−2^), fisheries stock status, total Fishery Management Plan interventions (i.e., modifications such as amendments, frameworks, motions, specifications, and addendums), and percentage of EEZ permanently protected from fishing as of 2017. Human population density values were derived from NOAA Digital Coast US Census decadal data available within coastal counties from the past three decades (i.e., 1990, 2000, 2010 censuses). Coastal counties are defined by NOAA and the US Census Bureau as those counties where at least 15% of a county’s total land area is located within the US coastal watershed^63^.

Population values were summed for all coastal counties within a given region and divided by county area. Total shelf area per region was calculated using NOAA Office of Coast Survey US maritime boundaries and limits spatial shapefile data^64^. Fishing effort (kilowatt sea days) was obtained from Watson^65^. Each managed US fisheries stock was examined for its June 2017 overfishing, overfished, or unknown status as reported for NOAA’s Fish Stock Sustainability Index (FSSI) and non-FSSI stocks^66^, and totals and proportions of stocks of a given status were summarized per region. All regional states marine fisheries commissions and federal FMPs and fishing regulations were examined for the total number of modifications it had undergone since its original release (as of 2017), and all values were summed per region. Finally, the total spatial extent of marine protected areas where commercial and/or recreational fishing is prohibited was tallied per region using NOAA’s Marine Protected Area Inventory (https://marineprotectedareas.noaa.gov/dataanalysis/mpainventory/). Afterward, the percent coverage of areas where commercial and/or recreational fishing is permanently prohibited was calculated relative to the EEZ of a given region (as of 2017).

Additionally, for international LMEs (n = 64), total fisheries landings and USD value (standardized to year 2017) were obtained from Watson^65^ and examined for the years 1998–2014 for each LME as related to primary productivity. LME primary productivity values were obtained as noted above for US LMEs. Relationships among LME productivity, fisheries landings, and USD value were cumulatively examined at continental and global scales.

## Supplementary Information

Supplementary Figures.

## Data Availability

The raw data from Watson^65^ are publicly available or available by request. All data and processed data generated or analysed during the current study are available upon request to the corresponding author.
