# Supplementary material for: Primary production ultimately limits fisheries economic performance
Source: Sci Rep. 2021 Jun 16;11:12154. doi: 10.1038/s41598-021-91599-0 (PMC8209017; doi:10.1038/s41598-021-91599-0)
Supplement: Supplementary file 1 — Supplementary Figures. [file 41598_2021_91599_MOESM1_ESM.docx]

Scientific Reports

Supplementary Materials for

**Primary production ultimately limits fisheries economic performance**

Anthony R. Marshak, Jason S. Link

*Corresponding author. Email: [tony.marshak@noaa.gov](mailto:tony.marshak@noaa.gov)

**This file includes:**

Figures S1 to S3


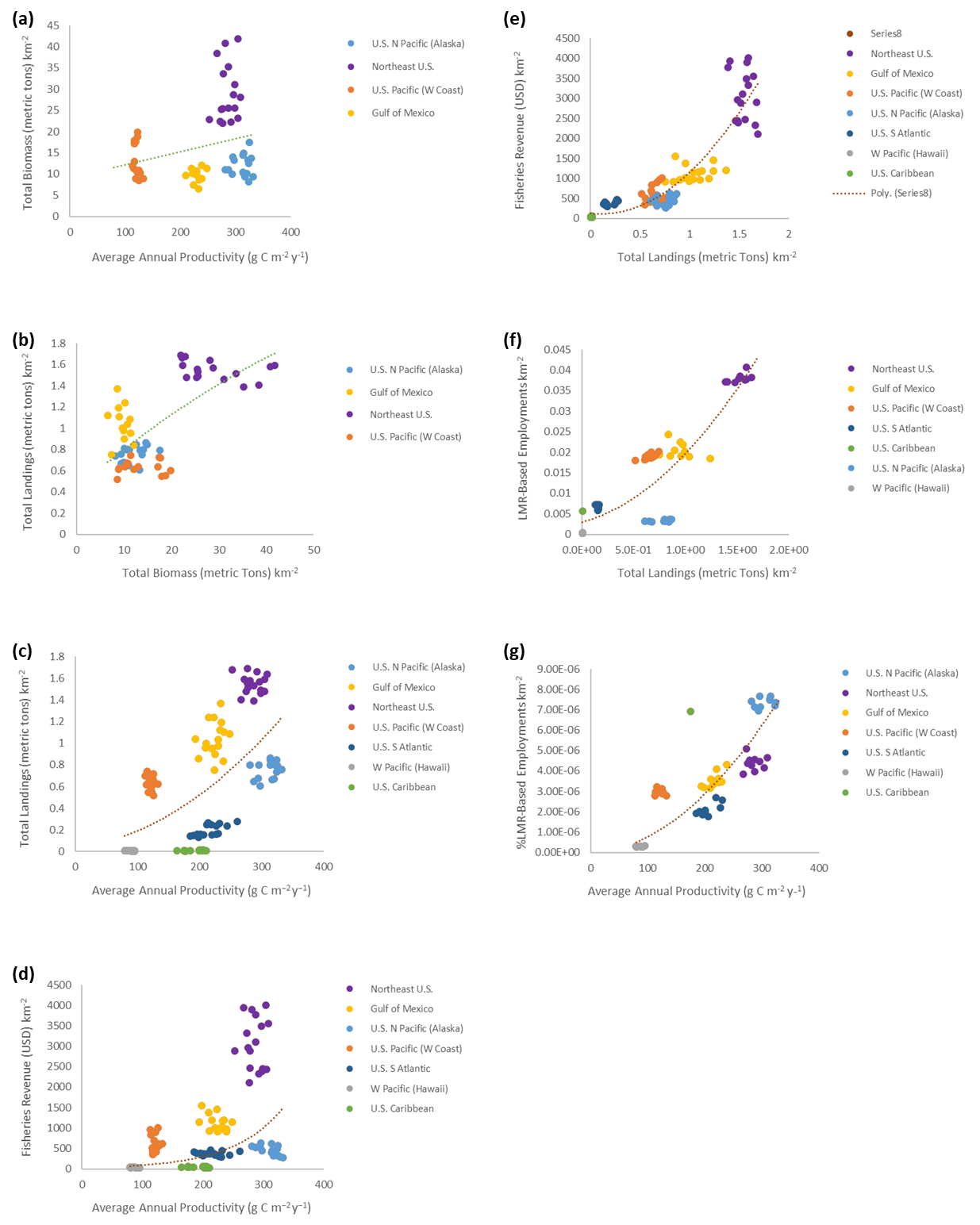


Figure S1.

Relationships between (**a**) average annual primary productivity (g C m-2 y-1) and per-area surveyed fish and invertebrate biomass (metric tons km^-2^; Years 1998-2014; y=0.302x + 9.2298; r^2^=0.066; p<0.0001); (**b**) per-area surveyed fish and invertebrate biomass (metric tons km^-2^) and total fisheries landings (metric tons km^-2^; Years 1998-2014; y=-0.0002x^2^ + 0.0384x + 0.4367; r^2^=0.492; p<0.0001); (**c**) average annual productivity (g C m^-2^ y^-1^) and total fisheries landings per area (metric tons km^-2^; years 1998-2014; y=9*10^-6^ x^2^ + 0.0007x + 0.0361; r^2^=0.367; p<0.0001); (**d**) average annual productivity (g C m^-2^ y^-1^) and total fisheries revenue (USD km^-2^; Years 1998-2014; y= 27.385e^0.012x^; r^2^=0.340; p<0.0001); (**e**) total fisheries landings per area (metric tons km^-2^) and total fisheries revenue per area (USD km^-2^; Years 1998-2014; y=1311.4x^2^ - 280.96x + 126.74; r^2^=0.860; p<0.0001); (**f**) total fisheries landings per area (metric tons km^-2^) and total living marine resource (LMR)-based employment (km^-2^; Years 2005-2014; y=0.01x^2^ + 0.0067x + 0.003; r^2^=0.782; p<0.0001); (**g**) average annual productivity (g C m^-2^ y^-1^) and percentage of LMR-based employment within total ocean economy per unit area (km^-2^; years 2005-2014; y=1*10^-10^x^1.9087^; r^2^=0.718; p<0.0001).


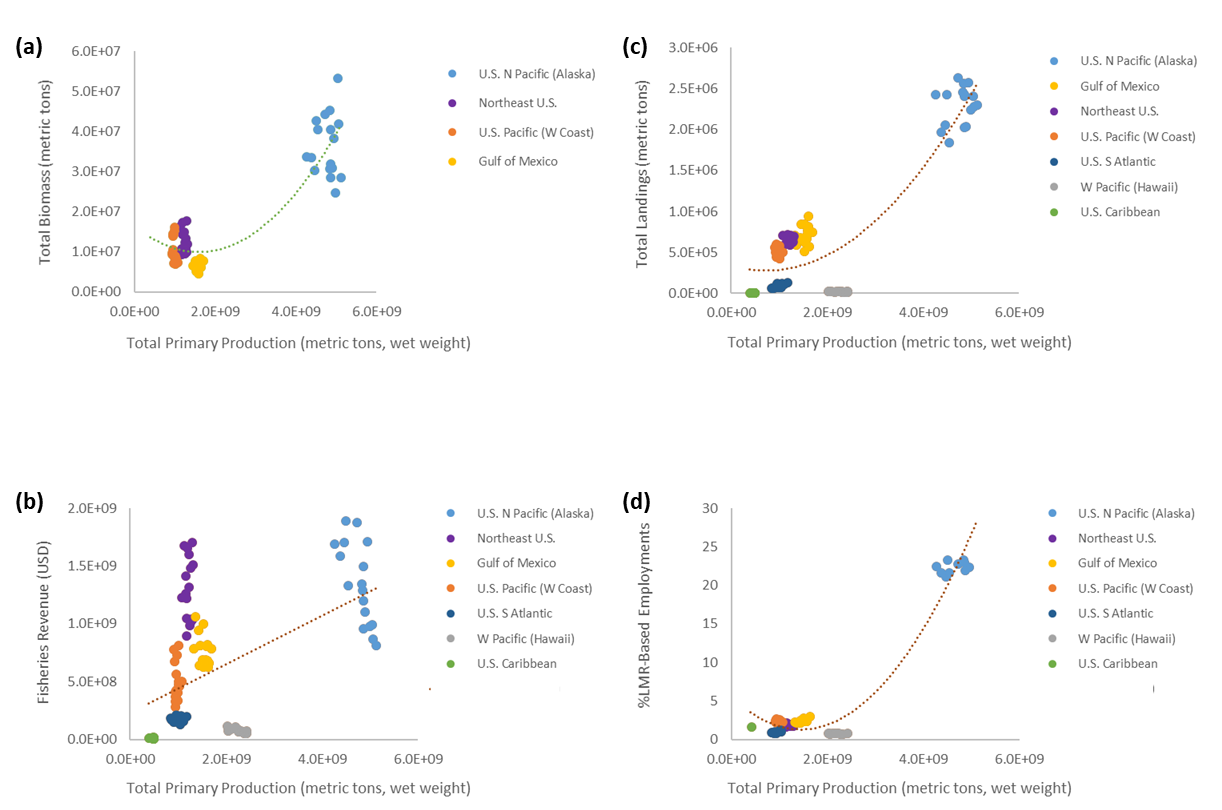


Figure S2.

Relationships between (**a**) total primary production (metric tons) and total surveyed fish and invertebrate biomass (metric tons; Years 1998-2014; y=3*10^-12^ x^2^ – 0.0081x + 2*10^7^; r^2^=0.833; p<0.0001); (**b**) total primary production (metric tons) and total fisheries revenue (USD; y=0.2101x + 2*10^8^; r^2^=0.265; p<0.0001); (**c**) total primary production (metric tons) and total fisheries landings (metric tons) throughout all major U.S. regions (years 1998-2014; y=1*10^-13^ x^2^ – 0.0002x + 346519; r2=0.792; p<0.0001); (**d**) total primary production (metric tons) and percentage of living marine resource (LMR)-based employment within total ocean economy (Years 2005-2014; y=2*10^-18^ x^2^ – 6*10^-9^x + 5.5146; r^2^=0.962; p<0.0001).


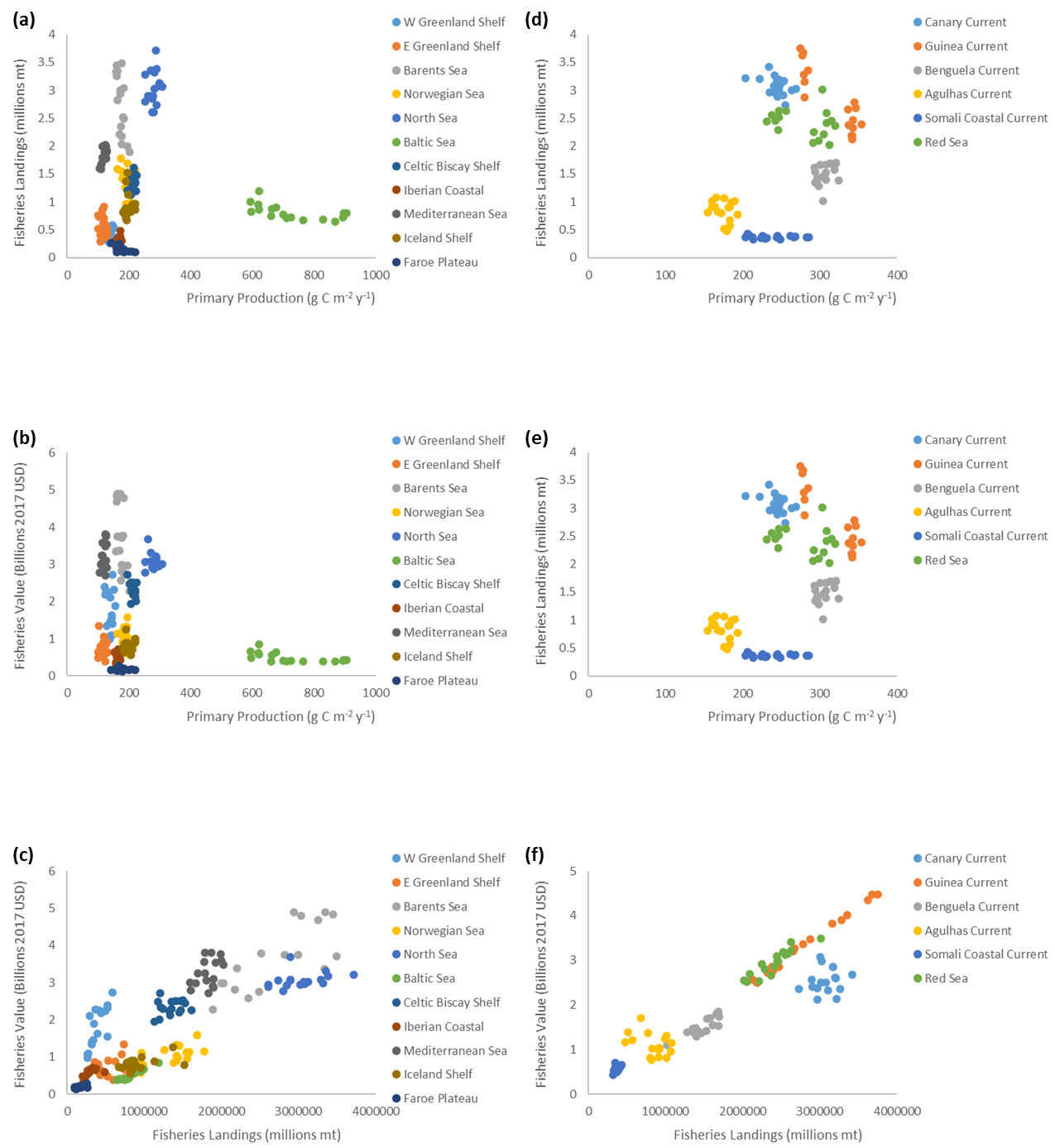


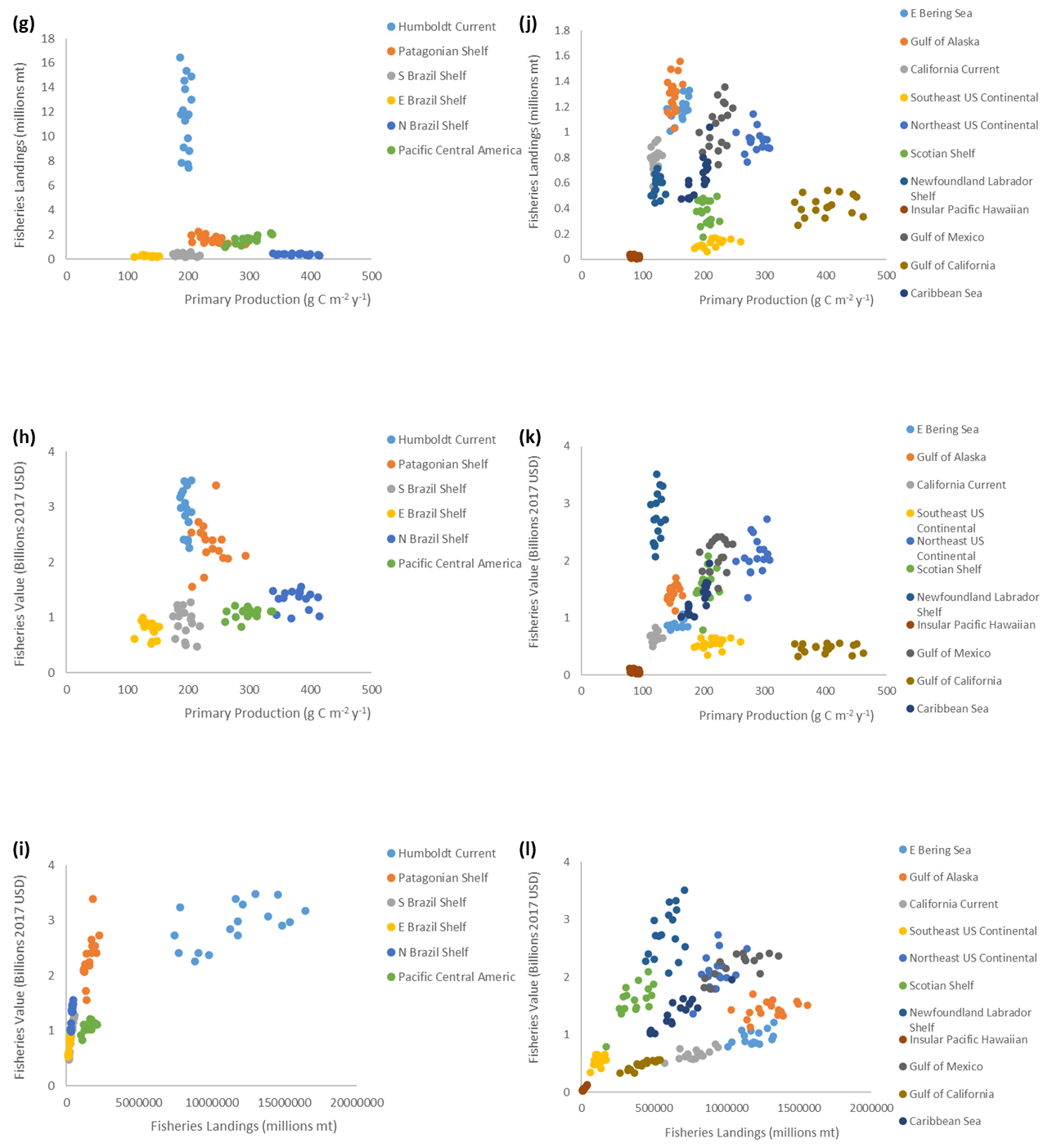

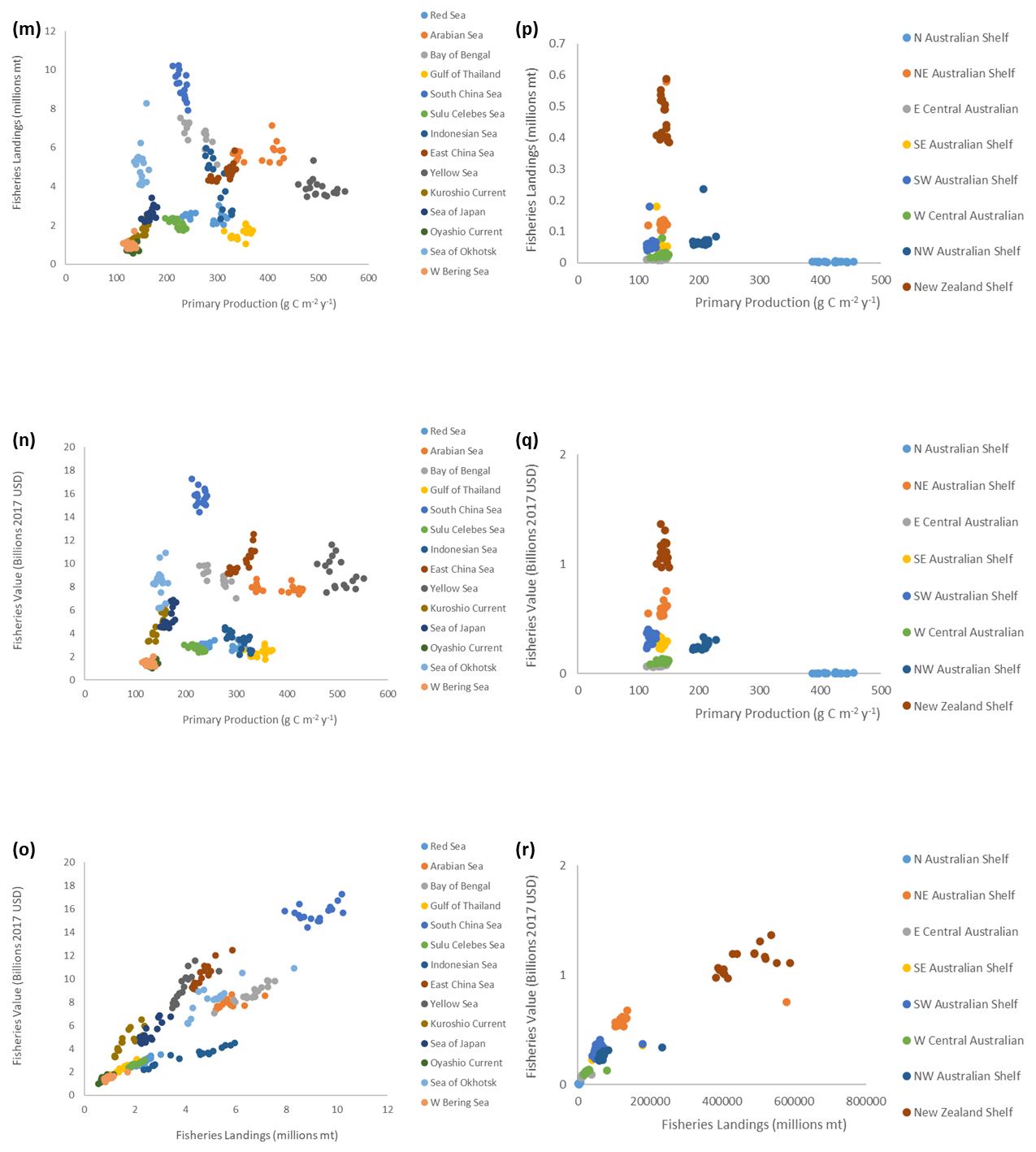


Figure S3.

For the eleven identified European Large Marine Ecosystems, relationships between (**a**) average annual primary productivity (g C m^-2^ y^-1^) and total fisheries landings (metric tons, mt); (**b**) average annual primary productivity and fisheries value (2017 USD); and (**c**) total fisheries landings and fisheries value. For the six identified African Large Marine Ecosystems, relationships between (**d**) average annual primary productivity (g C m^-2^ y^-1^) and total fisheries landings (metric tons, mt); (**e**) average annual primary productivity and fisheries value (2017 USD); and (**f**) total fisheries landings and fisheries value. For the six identified South American Large Marine Ecosystems, relationships between (**g**) average annual primary productivity (g C m-2 y-1) and total fisheries landings (metric tons, mt); (**h**) average annual primary productivity and fisheries value (2017 USD); and (**i**) total fisheries landings and fisheries value. For the eleven identified North American Large Marine Ecosystems, relationships between (**j**) average annual primary productivity (g C m^-2^ y^-1^) and total fisheries landings (metric tons, mt); (**k**) average annual primary productivity and fisheries value (2017 USD); and (**l**) total fisheries landings and fisheries value. For the 14 identified Asian Large Marine Ecosystems, relationships between (**m**) average annual primary productivity (g C m^-2^ y^-1^) and total fisheries landings (metric tons, mt); (**n**) average annual primary productivity and fisheries value (2017 USD); and (**o**) total fisheries landings and fisheries value. For the eight Large Marine Ecosystems identified for Oceania (Australia and New Zealand), relationships between (**p**) average annual primary productivity (g C m^-2^ y^-1^) and total fisheries landings (metric tons, mt); (**q**) average annual primary productivity and fisheries value (2017 USD); and (**r**) total fisheries landings and fisheries value. Years cover 1998-2014.
